# Sources of Human Campylobacteriosis Cases in Estonia and the Genomic Associations with Broiler Chicken Meat Isolates

**DOI:** 10.3390/pathogens15050539

**Published:** 2026-05-16

**Authors:** Ilijana Ivanov, Hanna Katriina Takkinen, Johanna Takkinen, Mati Roasto, Mihkel Mäesaar

**Affiliations:** 1 Chair of Veterinary Biomedicine and Food Hygiene, Institute of Veterinary Medicine and Animal Sciences, Estonian University of Life Sciences, F. R. Kreutzwaldi 56/3, 51006 Tartu, Estoniamati.roasto@emu.ee (M.R.); 2 Independent Researcher, Juhonpolku 8, 15540 Villähde, Finland

**Keywords:** *C. jejuni*, *C. coli*, cgMLST, AMR, source attribution

## Abstract

This study used three complementary datasets to investigate the relationship between human *Campylobacter* infections in Estonia and potential sources. A targeted dataset of 15 *C. jejuni* genomes with overlapping sequence types from human cases and broiler chicken meat was analysed using genotyping and in silico antimicrobial resistance profiling, alongside 20 human isolates for source attribution. Additionally, 12,111 isolates were analysed to provide population-level context. The core genome multilocus sequence typing showed a high similarity (less than three allelic differences) between the human and broiler isolates of ST122, ST464, and ST7355, indicating poultry as a likely source, whereas ST9882 was more divergent (13–18 allelic differences). The resistance profiles were consistent within ST122, ST464, and ST7355, and all were resistant to ciprofloxacin, nalidixic acid, ampicillin, and tetracycline, while ST9882 additionally exhibited aminoglycoside (streptomycin) resistance. The source attribution linked 77.8% of the human cases to chicken and 22.2% to cattle. A novel genotype, ST11001, was identified in humans and attributed to cattle source, while *C. coli* isolates were linked to birds and sheep. Poultry dominated the larger dataset (87.3%). Gastroenteritis was the predominant clinical presentation (98.5%), whereas ST22 and ST122 were associated with Guillain–Barré syndrome. These findings support poultry as a major reservoir of human *Campylobacter* infections and highlight the need for coordinated cross-border surveillance.

## 1. Introduction

Campylobacteriosis was the most frequently reported foodborne gastrointestinal disease in the European Union (EU) in 2022 and 2023, with 148,181 cases reported in 2023 alone [1,2]. The disease is primarily caused by *Campylobacter jejuni* and *C. coli*, with *C. jejuni* responsible for nearly 90% of the cases in the EU [1]. Despite increasing investigations into the sources of infection [3], the survival mechanisms of *Campylobacter* in various environments remain poorly understood [4].

*C. jejuni* has been isolated from numerous sources, including wild and domestic animals, livestock, and the environment [5]. However, poultry, especially broiler chickens, represent the most significant reservoir for human infections [1,6,7,8,9]. The pathogen can persist in farm equipment, poultry houses, environmental waters, and faeces for several days [10,11,12], and although it cannot replicate outside a host, it remains viable under diverse environmental conditions [13]. The ingestion of contaminated food or water leads to symptoms such as diarrhoea, fever, and abdominal pain, with potential complications [14,15].

The leading risk factor for human campylobacteriosis is the consumption of raw or undercooked poultry, especially chicken [1,9,16,17,18]. High levels of bacterial shedding by colonised poultry and cross-contamination in the poultry supply chain and during food preparation contribute to transmission [19,20,21,22].

Multilocus sequence typing (MLST) has been a cornerstone for long-term epidemiological studies, identifying slowly evolving genetic lineages [23], but its resolution is limited by its reliance on only seven loci. In contrast, whole-genome sequencing (WGS), especially core genome MLST (cgMLST), allows for higher-resolution strain differentiation [24]. The Oxford cgMLST scheme includes 1343 loci for *C. jejuni* and *C. coli* [25], though the number of loci varies based on the scheme design and the genomic diversity [26]. Allelic differences (ADs) in cgMLST are used to quantify genetic similarity. While no universal AD threshold exists, cutoffs between 4 and 20 have been previously used in outbreak investigations to infer relatedness [27,28,29].

WGS and cgMLST have enabled the detection of outbreak clusters and source attribution by linking clinical isolates to those from food and animals [25,28]. Several approaches to source attribution exist, including comparative exposure assessment, epidemiological interviews, and microbial subtyping [30,31]. Microbial subtyping compares WGS data from human isolates to potential source isolates, though its accuracy depends on source sampling coverage [30].

Machine learning approaches now enhance microbial subtyping by predicting infection sources from cgMLST or k-merised WGS data. Recent models, such as those using extreme gradient boosting, achieved accuracy up to ~85% with cgMLST data [32]. These tools offer robust attribution even for genetically novel strains.

We hypothesise that *Campylobacter* strains from Estonian clinical cases and broiler meat sold in Estonia are genetically related, indicating poultry as a major infection source. This study evaluates the relationships using cgMLST allelic distances, antimicrobial resistance (AMR) profile concordance, and source attribution probabilities. It focuses on the key genotypes (ST122, ST464, ST7355, and ST9882) to explore the genomic connections between the isolates of human and broiler chiken meat source. By integrating the cgMLST data with machine learning-based source attribution, the analysis identifies likely reservoirs contributing to human campylobacteriosis in Estonia while revealing genome-level links among the strains.

## 2. Materials and Methods

This study used three complementary datasets to investigate the relationship between human *Campylobacter* infections in Estonia and their potential sources. A targeted dataset of 15 *C. jejuni* genomes, selected based on the presence of overlapping MLST sequence types (ST122, ST464, ST7355, and ST9882) identified both in Estonian human clinical cases and Lithuanian broiler chicken meat, was used for the genomic relatedness (cgMLST) and in silico AMR analyses. A second dataset of 20 Estonian human clinical isolates was analysed for source attribution using a machine learning approach, which partially overlaps with the first dataset. A third dataset of 12,111 *Campylobacter* isolates, excluding the isolates analysed in this study, was used for data mining to provide a population-level context.

The analyses were conducted sequentially to assess genomic similarity (cgMLST), reflecting genetic relatedness, and concordance in AMR profiles as functional supporting evidence. Together, these provide complementary evidence consistent with a shared origin. This was followed by source attribution analysis to predict campylobacteriosis sources and provide probabilistic support for the associations identified in the genomic analyses. Finally, data mining was performed to contextualise these findings within a broader epidemiological framework and to assess the distribution of genotypes and their associations with potential sources at the population level.

### 2.1. Genomic Association Analysis

The genome-level association analysis was conducted using 15 previously sequenced *C. jejuni* genomes (Table 1) representing four genotypes, each including at least one human clinical isolate from Estonia and one broiler chicken meat isolate [33,34]. As described by Tedersoo et al. [33], the genotypes were assigned using the *C. jejuni*/*coli* public multilocus sequence typing database (*C. jejuni*/*coli* PubMLST). These isolates were selected based on the presence of overlapping sequence types in both human and poultry sources. This analysis is exploratory due to the limited number of available genomes.

To assess the genomic relatedness between the four STs identified among the 15 isolates from human and broiler chicken meat origin, a cgMLST analysis was performed for ST122, ST464, ST7355 and ST9882 using the genome comparator tool from the bacterial isolate genome sequence *C. jejuni*/*coli* PubMLST database [34,35]. The first version of the *C. jejuni*/*coli* cgMLST scheme (1343 loci) was applied with the default settings [34]. The pairwise distance matrices were calculated between the human and chicken isolates using the cgMLST ADs for each ST. Differences in less than five loci were considered indicative of potential clonal relationships, reflecting a conservative cutoff for recent transmission [28].

### 2.2. In Silico Antimicrobial Resistance Phenotype Analysis

The in silico AMR analyses were conducted on the same set of 15 genomes used in the genomic association analysis to evaluate the AMR profile concordance with the cgMLST-based similarity.

The analyses were performed using the WGS data on the Technical University of Denmark’s Centre for Genomic Epidemiology platform. All of the 15 genomes downloaded from the *C. jejuni*/*coli* PubMLST database [34] were analysed using ResFinder (v4.7.2) software with the ResFinder (v2.5.1) and PointFinder (v4.1.1) databases, applying the default parameters and selecting *C. jejuni* as the species [36,37].

### 2.3. Source Attribution Analysis

For the source attribution, 20 *Campylobacter* spp. human isolates (18 *C. jejuni* and 2 *C. coli*) from Estonian enteric patients (2017–2019) were obtained from the *C. jejuni*/*C. coli* PubMLST database [34], originally sequenced by Tedersoo et al. [33].

As described by Arning et al. [32] the first version of the *C. jejuni* and *C. coli* Oxford cgMLST schema was used to extract the allelic profiles from 20 Estonian human *Campylobacter* isolates obtained from the *C. jejuni*/*coli* PubMLST database using the export dataset plugin with the default settings [34]. These cgMLST profiles served as the input for the aiSource algorithm for source attribution, relying on a machine learning model trained on cgMLST profiles from multiple host reservoirs [32]. While the model has achieved reported accuracy of up to ~85%, its predictions are inherently dependent on the composition and representativeness of the training dataset and may be less reliable for rare or previously unobserved STs. Therefore, the final predicted source corresponds to the one with the highest probability among all the included sources.

### 2.4. Data Mining

A total of 12,111 isolates of *C. jejuni* (*n* = 12,032) and *C. coli* (*n* = 79), originating from the PubMLST database [34], were used for the data mining analysis to determine the sources and the disease outcomes. The dataset was constructed based on the STs included in the source attribution analysis, thereby enabling the comparison of genotype distribution and source associations across a larger population. This approach also allowed the contextualisation of the genotypes included in the genomic relatedness and AMR analyses within a broader epidemiological framework.

The data from the *C. jejuni*/*coli* PubMLST database [34] was collected on 8 January 2025. First, the database search step included the following procedures: (1) field filter: ST (MLST) was used with MLST genotype information obtained from the 20 *Campylobacter* spp. human isolates; (2) provenance fields: “all” and schemes: “typing” were selected. This resulted in an initial dataset, which included 12,197 isolates and 12,317,003 data fields (excluding headers). Second, the dataset cleaning step was performed by applying the following steps: (1) removed the isolate records which did not have species information; (2) removed the isolate records which indicate contamination from “comments” column; (3) removed the isolates which had multiple genotype values in the “ST (MLST)” column; (4) removed the isolate records (*n* = 20) included in the present study; (5) removed the conflicting species of *C. coli* when ST and species did not match; (6) deleted the columns with unnecessary data. The cleaned dataset included 12,111 isolates and 104,026 data fields for further source and epidemiological analyses.

The isolates were grouped by ST and categorised by source: beef offal/meat, broiler environment, calf, cat, cattle, cattle faeces, chicken, chicken offal/meat, cow’s milk, dairy, dog, duck, environmental waters, farm environment/slurry, goat, goose, human blood culture/stool/unspecified, lamb/offal/meat, other animals, pig, pork offal/meat, potable water, rabbit, sheep/faeces, turkey/offal/meat, vegetables, wild bird, and unknown. The disease-related categories included carrier, gastroenteritis, Guillain–Barré syndrome, systemic disease, and unknown.

The sources were combined into broader categories: poultry (broiler environment, chicken, chicken offal/meat, duck, goose, turkey, turkey offal/meat), pet (cat, dog), environment and wild bird (environmental waters, farm environment, farm slurry, potable water, wild bird), human (blood culture, stool, unspecified), other animal (other animals, rabbit), vegetables, and farm animals (beef offal/meat, calf, cattle, cattle faeces, cow’s milk, dairy products, goat, lamb/offal/meat, pig, pork offal/meat, sheep/faeces). The data mining was performed using Microsoft Excel (Redmond, WA, USA).

### 2.5. Statistical Analyses

The statistical analysis was performed using Microsoft Excel and descriptive statistics. The 95% confidence interval (95% CI) for a proportion, incorporating a continuity correction, was calculated using the VassarStats website [38,39,40].

## 3. Results

### 3.1. Genomic Relatedness (cgMLST)

**Figure 1:**
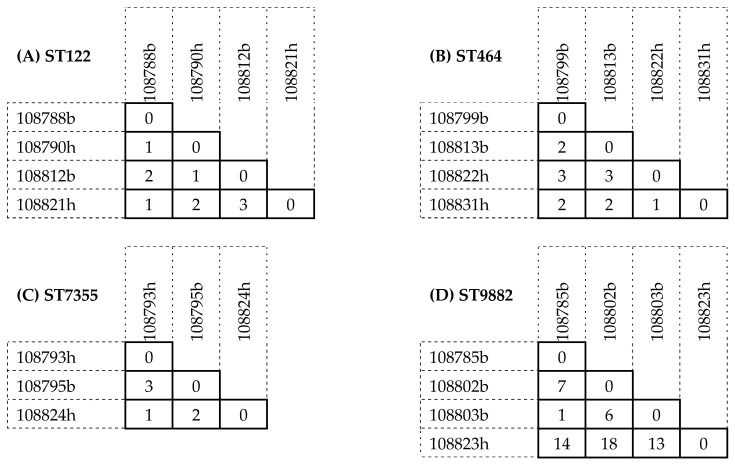
Pairwise core genome multilocus sequence typing allelic difference (<5 allelic differences potentially clonal) matrices for the following sequence types (STs): (**A**) ST122, (**B**) ST464, (**C**) ST7355 and (**D**) ST9882. For each isolate, the *C. jejuni*/*coli* public multilocus sequence typing database identification number is given together with the source Lithuanian broiler chicken meat (b) and Estonian clinical human isolate (h).

The cgMLST genomic similarity analysis showed a high allele similarity (less than five ADs) between the Estonian human clinical isolates and the Lithuanian broiler chicken meat isolates for the *C. jejuni* genotypes ST122 (Figure 1A), ST464 (Figure 1B), and ST7355 (Figure 1C). Conversely, while the chicken meat isolates of ST9882 were closely related, the human isolate in this genotype was more divergent, with 13–18 ADs compared to the chicken isolates (Figure 1D).

### 3.2. Antimicrobial Resistance Profiles

The in silico WGS analysis revealed consistent AMR profiles within each *C. jejuni* genotype regardless of origin. Specifically, the genotypes ST122, ST464, and ST7355 were resistant to ampicillin, ciprofloxacin, nalidixic acid, and tetracycline, while ST9882 exhibited resistance to these agents as well as aminoglycosides class (Table 2).

Quinolone resistance (ciprofloxacin and nalidixic acid) was associated with the *gyrA* mutation (Thr86Ile), whereas resistance to tetracycline, ampicillin, and streptomycin was mediated by the *tet(O/32/O)*, *blaOXA-61*, and *ant(6)-Ia* genes, respectively. The genotypic and phenotypic AMR profiles were in agreement with those reported by Tedersoo et al. [41].

### 3.3. Source Attribution

The 20 human isolates analysed for source attribution represented 13 MLST genotypes (Table 3), including one novel *C. jejuni* genotype, ST11001. Among the 18 *C. jejuni* isolates from Estonian patients, 22.2% (95% CI: 7.4–48.1%) were attributed to cattle and 77.8% (95% CI: 51.9–92.6%) to chicken. The wide confidence intervals reflect a limited sample size.

The two *C. coli* isolates were linked to bird and sheep sources (Table 3). The average main source prediction probability of all (*n* = 20) the Estonian human *Campylobacter* isolates was 0.732.

### 3.4. Data Mining Results

#### 3.4.1. Source Distribution

Table 4 provides a focused overview of the four MLST genotypes shared between the human and broiler chicken isolates without combining the source categories, whereas Table 5 presents a broader data mining-based overview of all the genotypes with combined source categories. As shown in Table 4, the sample origin was reported for 90.0% of the isolates, with 52.3% originating from humans. When excluding human and unknown sample origin, chicken was the dominant source with 93.2%.

The data mining of the 12,111 isolates across the genotypes assigned sources to 85.3% (*n* = 10,328) of the samples (Table 5). Humans accounted for 44.4% of the isolates, followed by poultry and farm animals combined at 39.7%. Environment and wild bird, other animal, pet, and vegetable sources each represented approximately 0.5%, 0.4%, 0.3%, and <0.1%, respectively (Table 5).

#### 3.4.2. Genotype Distribution Across Sources

Six sources harboured various *C. jejuni* and *C. coli* genotypes (Table 6). ST464 was found in all six sources; ST22, ST50, ST122, ST353, and ST572 in five; ST824 and ST1595 in four and three; ST429 and ST1624 in two; and ST7355 only in poultry. ST9882 was unique to humans, while the novel ST11001 was previously unreported (Table 5 and Table 6). Overall, the genotypes were most frequently associated with poultry (87.3%), farm animals (9.7%), and environment and wild birds (1.2%), with other sources each contributing less than 2.0% combined (Table 6).

#### 3.4.3. Clinical Outcomes

Among the 5372 human isolates with specific STs, disease information was available for 56.5% of the cases. Most of the cases with clinical information (98.5%) presented with gastroenteritis, while 0.5% were asymptomatic carriers (Table 7).

A total of 32 cases were linked to either Guillain–Barré syndrome (*n* = 26) or systemic diseases (*n* = 6). Guillain–Barré syndrome was primarily associated with the genotypes ST22 (25 cases) and ST122 (one case). These genotypes, along with ST50 and ST353, were also linked to systemic diseases (Table 7). However, nearly half of the cases lacked disease outcome data.

#### 3.4.4. Epidemiological and Demographic Characteristics

Table 8 and Table 9 provide a focused overview of the MLST genotypes shared between the Estonian human clinical isolates and broiler chicken meat (ST122, ST464, ST7355, and ST9882), rather than all the genotypes identified in the dataset.

The epidemiological classification (Table 8) showed that most of the cases were sporadic (98.4%), with a few linked to carriers, hospital inpatients, and outbreaks (*n* = 11). However, 46.2% of the human isolates lacked epidemiological classification data (Table 8).

Gender data were available for 53.9% of the human clinical isolates, showing a near-equal distribution: 47.5% female (95% CI: 43.7–51.3) and 52.5% male (95% CI: 48.7–56.3) (Table 9).

The average patient age ranged from 25 to 34 years across the STs, with mean ages of 6.1, 36.3, and 70.4 years in the 0–14, 15–64, and 65+ age groups, respectively. Most of the cases were of the genotype ST122 (Table 9). The 15–64 age group was most affected (170 cases), followed by children under 14 and the elderly, mainly linked to ST122 and ST464.

No data on epidemiology (Table 8), disease outcome (Table 7), or demographics (Table 9) were available for ST9882.

## 4. Discussion

Although *Campylobacter* infections are typically considered sporadic [42] and may arise from multiple sources [5,32], advances in WGS and machine learning-based source attribution suggest that genomic links between human and non-human isolates are more common than previously recognised [27,32,43,44,45]. In Estonia, Tedersoo et al. [33] identified four overlapping *C. jejuni* genotypes (ST122, ST464, ST7355, ST9882) among sporadic human cases and broiler chicken meat products of Lithuanian origin sold at retail. Building on these findings, the present study applied cgMLST to further resolve genomic relatedness between human and poultry isolates and to evaluate poultry as a potential reservoir for human infection [9].

EU member states annually report foodborne outbreak data to the European Food Safety Agency (EFSA), providing regional insights into outbreak trends by pathogen. In 2023, *Campylobacter* was the second most frequently reported cause of foodborne outbreaks in the EU after *Salmonella*. Despite a 10% decline in the total number of outbreaks, *Campylobacter*-related case numbers and hospitalisations remained stable [2]. In 2023, 15 strong-evidence outbreaks associated with broiler meat and related products were reported in Denmark, France, Lithuania, Poland, and Spain. *Campylobacter* and broiler meat (and products thereof) ranked fifth among the top ten pathogen–food vehicle combinations linked to outbreaks in the EU [2]. A typing-based surveillance in Denmark in 2019 identified a prolonged outbreak caused by a genomically stable ST122 strain, resulting in 91 confirmed cases and an estimated 700 infections overall. The outbreak source was traced to a single poultry slaughterhouse and at least one farm [29]. These findings support the assumption that *Campylobacter* strains can persist long-term in the poultry production chain and align with our current findings consistent with poultry as a likely source of ST122 infections in Estonia (Figure 1A). Unfortunately, no metadata beyond the isolation year were available for the Estonian human isolates at the time of this study.

Several European studies are in agreement with our results, particularly the overlap of STs between human and poultry sources. A Polish study analysing over 600 *C. jejuni* isolates found shared STs, including ST464 and ST122, in human and poultry samples [46]. A case-control study in Luxembourg from 2010 to 2013 combining MLST and risk factor analysis detected ST464 and ST122 in human isolates, though they were not among the five most prevalent STs [47]. In France, 76% of broiler meat samples were contaminated with *Campylobacter* spp. [48]. Among the 175 isolates typed by MLST, ST464 ranked in the three most common STs, confirming its widespread presence in poultry products [48]. Finally, a three-year longitudinal study at a Swiss poultry abattoir documented the recurrent presence of ST122 and ST464 [49]. Our data mining revealed that the genotypes ST464 and ST122 have been identified across multiple sources but are predominantly associated with chicken (Table 4). Both are primarily linked to sporadic gastroenteritis cases, though notable differences exist. ST464 has also been connected to outbreaks (Table 8), whereas ST122 has been isolated from patients with more severe manifestations, including Guillain–Barré syndrome and systemic infections (Table 7). The average age of the patients infected with ST464 and ST122 was similar. However, ST464 was more frequently isolated from males than females (Table 9). This male predominance may be influenced by sex-specific differences in host susceptibility or immune response, which have been shown to contribute to higher incidence and colonization rates of *Campylobacter* in males across age groups, independent of behavioural factors [50,51]. Physiological or genetic differences [51] may also contribute to the variation in clinical presentation or the likelihood of asymptomatic carriage, consistent with the patterns observed here for ST464 infections.

There has been a clear rise in resistance to ciprofloxacin and nalidixic acid in Europe over the last two decades, with over half of the *Campylobacter* spp. isolated from broiler meat at the retail level showing resistance to fluoroquinolones worldwide [52]. The 2022–2023 EU AMR summary report documented a significant increase in ciprofloxacin resistance in both poultry-associated and human *Campylobacter* isolates from 2014 to 2023, with the highest rates observed in Lithuanian human isolates. The fourth joint inter-agency EU report found a positive correlation between fluoroquinolone use in poultry and ciprofloxacin resistance levels in *C. jejuni* [53].

In Estonia, ciprofloxacin resistance due to the *gyrA* mutation was detected in over 60% of poultry *Campylobacter* isolates, especially in imported products, with even higher resistance proportions among human clinical cases [54,55]. Poultry meat imported from Lithuania and Latvia showed the highest AMR rates. Furthermore, according to Mäesaar et al. [54], approximately 7% of Estonian human *Campylobacter* spp. isolates exhibited multidrug resistance. These findings underscore the importance of integrated genomic and phenotypic AMR monitoring in human and non-human *Campylobacter* strains, considering the complex and regionally variable resistance mechanisms. Overall, the data supports a link between human foodborne *C. jejuni* infections from poultry sources, consistent with prior studies in the Baltic region [9,33,56,57] and across Europe [2].

The data mining revealed that nearly all the identified STs have been previously associated with human *Campylobacter* infections (Table 5). However, the genotype ST11001, identified by Tedersoo et al. [33], is a novel ST detected in humans for the first time globally (Table 3). Disease information was available for only 56.5% of the cases but provided valuable insights. Most of the cases reported gastroenteritis, the most common manifestation of campylobacteriosis [58], while a small number (0.3%) were asymptomatic carriers. Rao et al. [59] noted that asymptomatic *Campylobacter* excretion can continue for months following *Campylobacter*-related diarrhoea, potentially due to sustaining transmission.

The data mining study identified 26 Guillain–Barré syndrome cases: 25 linked to ST22 and one to ST122, representing ~20% of the *C. jejuni* genotypes from the Estonian patients included in the present study. Guillain–Barré syndrome’s association with *C. jejuni* is well established [60]. Linking specific genotypes to disease progression may enhance our understanding of host–pathogen interactions and inform targeted prevention and treatment strategies. Furthermore, six systemic infections were linked to ST50, ST353, ST22, and ST122.

The low infectious dose of *C. jejuni* [61], combined with multiple possible infection sources, complicates disease prevention and highlights the need for accurate source attribution. Although no isolates were directly linked to environmental sources, the environmental persistence of campylobacters contributes to transmission and bacterial spread [62].

Consistent with our results, the genotype ST464 has been linked to human infections in Latvia [57] and Lithuania [63], and it has also been detected in Lithuanian retail broiler products [64]. Similarly, ST122 associated with human infection has been reported in Lithuania [56,64]. While seven-gene MLST enables the classification of *Campylobacter* isolates into STs, its limited resolution has led to the adoption of cgMLST for higher-resolution cluster detection [28,49]. This study is the first in the Baltic States to demonstrate the strong genomic links of ST464 (Figure 1A) and ST122 (Figure 1B) between imported Lithuanian poultry products and Estonian human cases via cgMLST. Despite the high genomic diversity of *Campylobacter* revealed by cgMLST, certain STs, notably ST122 and ST464, repeatedly occur in both human infections and the poultry production chain [26,29,46,49].

The metadata for the *C. jejuni* genotype ST7355 were limited, but it has been previously identified in sporadic human cases (Table 7 and Table 8) and poultry sources (Table 4). Several studies confirm its presence in human infections and poultry, underlining its zoonotic potential [65,66,67]. Our finding of a high genomic similarity between an Estonian clinical isolate and a Lithuanian broiler meat isolate (Figure 1C) further supports an epidemiological link between human infections and poultry.

A key consideration in WGS-based genotyping is the threshold for genomic similarity between the isolates, which varies across methods [27]. Our cgMLST analysis showed that the human and broiler meat isolates of ST122, ST464, and ST7355 differed by only one to three alleles (Figure 1A–C), indicating a strong, likely clonal relationship within the strict cluster threshold (less than five ADs) defined by Joensen et al. [28]. In contrast, the ST9882 human isolates exhibited up to 18 ADs from Lithuanian broiler isolates, suggesting a more distant genetic relationship and a less likely recent common source.

The close genomic relatedness of the human *C. jejuni* ST122, ST464, and ST7355 isolates to Lithuanian broiler meat isolates, with a maximum of three ADs, supports poultry products as a probable source of human infections in Estonia [9]. This is consistent with the overlapping AMR profiles (Table 2). Although ST9882 shares the same resistance markers between the human and poultry isolates (Table 2), the greater genomic divergence (more than four ADs) indicates a weaker genetic link. However, compared to *Listeria monocytogenes*, *Campylobacter* undergoes frequent recombination, and these thresholds should therefore be interpreted with caution [68,69].

A major limitation of this study is the lack of accompanying epidemiological data, which prevents a more detailed assessment of a direct association between the genomic findings and the specific transmission events or the exposure history. Therefore, the relationships between the human and poultry *C. jejuni* isolates rest solely on the genomic comparisons and the AMR profiling. Incorporating detailed epidemiological data in future research would improve the attribution accuracy and clarify the potential transmission dynamics. The limited metadata, including the absence of travel, dietary, or clinical information, also constrained the interpretation. Given the cross-border poultry trade and the shared genotypes across the EU, coordinated cross-border efforts to improve *Campylobacter* source tracking are warranted.

The data mining revealed ST9882 has been previously found in only one human case (Table 5) without a known infection source. However, the PubMLST data are subject to sampling bias and uneven geographic representation, which may influence the observed distribution of rare genotypes. While this study did not find strong genomic links between the human and poultry isolates of ST9882. To our knowledge it is the first to implicate poultry as a possible source for this genotype.

The source attribution using the aiSource algorithm [32] identified poultry as the primary source for 14 *C. jejuni* strains, with cattle attributed for four. These findings agree with Mäesaar et al. [9], where poultry predominated as the source of Estonian clinical isolates, followed by cattle, and with Cody et al. [3], who reported poultry as the dominant with ruminants as a significant secondary source.

Seven of the nine genotypes linked to chicken showed equal or higher source attribution probabilities than the dataset average (Table 3), except for ST122 and ST429, which had lower than average probabilities. The data mining indicates that these STs are predominantly associated with poultry-related sources: ST50, ST122, ST353, ST429, ST464, and ST824 showed high attribution probabilities (88.4–98.0%), potentially reflecting multiple ecological niches within the poultry production and supply chain. aiSource yielded consistent chicken attribution probabilities ranging from 0.588 to 0.988. The presence of multiple sources and variable prediction probabilities suggests additional transmission contributors. A *Campylobacter* strain that is highly prevalent in each reservoir is not necessarily the one most frequently causing human infection, and vice versa [32]. For instance, Frosth et al. [70] described broiler colonisation via drinking water, which was polluted by nearby cattle, potentially explaining similar attribution probabilities across the different source categories.

ST22 was attributed primarily to farm animals (52.5%) but also linked to poultry, pets, other animals, environment, and wild birds (Table 6) in the data mining analyses. However, aiSource attributed two ST22 isolates to chicken with probabilities of 0.597 and 0.900 (Table 3). Since ST22 is associated with Guillain–Barré syndrome [71], this suggests the possibility of genotype-specific patient care approaches targeting the prevention of post-campylobacteriosis complications.

This study represents the first analysis of Estonian human *C. coli* isolates suggesting sheep and birds as likely sources, diverging somewhat from prior studies (Table 3). Previous studies using asymmetric island source attribution models for *C. coli* primarily identified poultry and ruminants (cattle, sheep) as the probable sources with swine, which was considered a secondary source, even when not found in our study due to the used model [18,72,73,74]. Using the STRUCTURE model, Sheppard et al. [72] reported sheep as the main source for *C. coli* infections, followed closely by poultry, with both contributing to human infections [3]. The two Estonian human *C. coli* isolates showed prediction probabilities of 0.557 for sheep (ST1595) and 0.622 for birds (ST1624), lower than the dataset averages (Table 3). The data mining associated ST1624 mainly with farm animals and ST1595 with poultry (81.0% and 60.9%, respectively) (Table 6). These differences likely reflect aiSource’s use of cgMLST data covering genome-wide variation [32]. Both the *C. coli* STs were linked to gastroenteritis by data mining. While only two *C. coli* isolates were analysed in the present study, future studies with larger sample sizes from different sources could clarify the source attribution of *C. coli* infections in Estonia.

The *C. jejuni* strains fell into 11 STs, with ST572 and the novel ST11001 attributed to cattle and others to chicken (Table 3). The attribution probabilities varied, some isolates reached 0.940 while others were lower, reflecting possible data gaps and unaccounted sources, which limit the aiSource algorithm. To improve the accuracy of novel genotypes, retraining the classifier with additional isolates from new sources is recommended [32]. This issue was notable for ST11001, which was assigned to cattle with only 0.360 probability (Table 3), likely because ST11001 was not included in the original training dataset [32]. Thus, its predicted source should be interpreted with caution.

ST572 had a 0.578 probability for cattle, below the dataset average (0.732) of all the main source prediction probabilities, followed by poultry at 0.443, to some extent consistent with the data mining showing poultry as the main source, then farm animals, environment, and wild birds. Wilson et al. [5] previously reported ST572 mainly in chicken, whereas Arning et al. [32] linked it more to sheep and cattle, highlighting potential temporal or geographical shifts in the source attribution.

ST9882 and ST11001 had no animal source attribution in the data mining. ST9882 includes only two strains, one human and one without source data (Table 5), while ST11001 was novel. Although, the attribution of this genotype was possible via aiSource.

While the PubMLST metadata provides isolate counts and sources, aiSource leverages genome-wide host-segregating variation to enhance the source attribution predictions [32,34]. A key limitation of aiSource, similar to all microbial subtyping models, is its dependence on the available source isolates [30]. Therefore, machine learning models require frequent updates. Their prediction accuracy depends on current, comprehensive training data and regular retraining. Attribution is also affected by sampling bias, geography, seasonality, and potential misclassification due to host transitions or generalist strains, especially between sheep and cattle [32]. Combining multiple attribution methods, as suggested by Pires et al. [30], may overcome the individual model limitations and improve the accuracy. Finally, genotype-specific control measures targeting the prevention of post-infection sequelae and systemic disease could reduce antimicrobial use, helping in the mitigation of AMR in *Campylobacter* spp. [75].

## 5. Conclusions

*Campylobacter* infections in Estonia are mainly sporadic, with poultry identified as the likely major source of human *C. jejuni* infections based on the available data. This is supported by the strong genomic relatedness between the Estonian human and Lithuanian broiler meat isolates. The high-resolution cgMLST analysis showed that the genotypes ST122, ST464, and ST7355 differed by only one to three alleles between the human and poultry isolates, indicating likely recent common origins or direct transmission. These genotypes also shared overlapping AMR profiles, reinforcing the poultry connection.

Genotype ST122 was linked to more severe clinical outcomes in the data mining analysis, including Guillain–Barré syndrome and systemic infections, while ST464 was associated with a male predominance in disease cases.

For *C. coli*, the source attribution based on only two isolates suggested sheep and wild birds as potential reservoirs, but this finding is highly uncertain and likely reflects stochastic variation rather than a true shift from the poultry- and cattle-associated sources reported previously. The novel genotype ST11001 was detected for the first time in humans but showed an uncertain source attribution due to the limited data.

The machine learning-based source attribution identified poultry as the likely source for most of the *C. jejuni* genotypes, with cattle as a secondary contributor. However, the variability in prediction probabilities and the limitations in the available metadata suggest unaccounted sources and highlight the need for regular retraining of the machine learning-based source attribution models.

Overall, these findings, together with previous studies, indicate that poultry is an important source of human *Campylobacter* infections in Estonia and that imported broiler meat may contribute disproportionately, consistent with its higher contamination levels at retail compared with domestic broiler products. These results emphasise the importance of integrated genomic surveillance combined with epidemiological data to improve source tracking, control transmission, and address AMR. Genotype-specific monitoring could also help target interventions for severe disease forms linked to specific strains or genotypes.

## Figures and Tables

**Table 1:** Information on the *C. jejuni* isolates included in the genomic relatedness analysis.

ST	Id	Country	Source	Year
ST122	108788	Lithuania	Chicken meat	2018
	108812	Lithuania	Chicken meat	2018
	108790	Estonia	Human	2018
	108821	Estonia	Human	2018
ST464	108799	Lithuania	Chicken meat	2018
	108813	Lithuania	Chicken meat	2019
	108822	Estonia	Human	2019
	108831	Estonia	Human	2019
ST7355	108795	Lithuania	Chicken meat	2019
	108793	Estonia	Human	2018
	108824	Estonia	Human	2018
ST9882	108785	Lithuania	Chicken meat	2019
	108802	Lithuania	Chicken meat	2019
	108803	Lithuania	Chicken meat	2019
	108823	Estonia	Human	2018

ST, sequence type of multilocus sequence typing; Id, *C. jejuni*/*coli* public multilocus sequence typing database identification number.

**Table 2:** Pattern of the whole-genome sequencing data based on in silico antimicrobial resistance phenotype prediction (gray squares) of the isolates belonging to four multilocus sequence type genotypes with *C. jejuni*/*coli* public multilocus sequence typing database identification number, together with the source Lithuanian broiler chicken meat (b) and Estonian human clinical (h) isolates.

Antimicrobial	108821h	108812b	108790h	108788b	108831h	108822h	108813b	108799b	108824h	108795b	108793h	108823h	108803b	108802b	108785b	Class
Gentamicin																Aminoglycoside
Streptomycin																Aminoglycoside
Ampicillin																Aminopenicillin
Erythromycin																Macrolide
Ciprofloxacin																Quinolone
Nalidixic acid																Quinolone
Tetracycline																Tetracycline
	ST122				ST464				ST7355			ST9882				

ST, sequence type.

**Table 3:** Isolate information, including species, sequence type, identification number and aiSource attribution predictions and probabilities. The source prediction is based on the highest probability (in underlined italics).

Information			Source Probabilities					
Species	ST	Id	Prediction	Cattle	Chicken	Environment	Bird	Sheep
*C. jejuni*	572	108809	cattle	*0.699*	0.088	0.000	0.174	0.039
		108816	cattle	*0.517*	0.443	0.000	0.001	0.039
		108818	cattle	*0.517*	0.443	0.000	0.001	0.039
	11001 ^a^	108830	cattle	*0.360*	0.138	0.073	0.252	0.177
	22	108825	chicken	0.382	*0.597*	0.000	0.002	0.019
		108828	chicken	0.069	*0.900*	0.000	0.002	0.028
	50	108789	chicken	0.235	*0.721*	0.001	0.008	0.035
		108827	chicken	0.214	*0.743*	0.000	0.022	0.021
	122	108790	chicken	0.214	*0.607*	0.000	0.000	0.179
		108821	chicken	0.224	*0.588*	0.000	0.000	0.188
	353	108826	chicken	0.010	*0.988*	0.001	0.001	0.001
	429	108817	chicken	0.352	*0.607*	0.001	0.003	0.036
	464	108822	chicken	0.032	*0.948*	0.001	0.013	0.006
		108831	chicken	0.032	*0.948*	0.001	0.013	0.006
	824	108814	chicken	0.028	*0.869*	0.021	0.003	0.078
	7355	108793	chicken	0.056	*0.940*	0.000	0.004	0.000
		108824	chicken	0.056	*0.940*	0.000	0.004	0.000
	9882	108823	chicken	0.023	*0.974*	0.000	0.001	0.001
*C. coli*	1624	108815	bird	0.033	0.061	0.239	*0.622*	0.045
	1595	108829	sheep	0.008	0.211	0.002	0.222	*0.557*

^a^, novel sequence type; ST, sequence type; Id, *C. jejuni*/*coli* public multilocus sequence typing database identification number.

**Table 4:** Distribution of isolation sources for four *C. jejuni* multilocus sequence typing genotypes: ST122, ST464, ST7355, and ST9882.

Source	ST122	ST464	ST7355	ST9882	Total (%)	SA (%)
Broiler environment	6	0	0	0	6 (0.2)	0.7
Calf	1	0	0	0	1 (<0.1)	0.1
Cat	1	1	0	0	2 (0.1)	0.2
Cattle	3	10	0	0	13 (0.5)	1.4
Cattle faeces	0	2	0	0	2 (0.1)	0.2
Chicken	131	359	1	0	491 (20.4)	54.1
Chicken offal or meat	49	306	0	0	355 (14.7)	39.1
Cow’s milk	2	0	0	0	2 (0.1)	0.2
Dairy products	0	1	0	0	1 (<0.1)	0.1
Dog	6	0	0	0	6 (0.2)	0.7
Duck	1	0	0	0	1 (<0.1)	0.1
Environmental waters	3	1	0	0	4 (0.2)	0.5
Goose	0	1	0	0	1 (<0.1)	0.1
Human blood culture	8	11	0	0	19 (0.8)	-
Human stool	617	509	9	1	1136 (47.2)	-
Human unspecified	44	60	0	0	104 (4.3)	-
No information	62	175	2	1	240 (10.0)	-
Other animal	3	3	0	0	6 (0.2)	0.7
Pig	0	3	0	0	3 (0.1)	0.3
Rabbit	0	1	0	0	1 (<0.1)	0.1
Turkey	2	5	0	0	7 (0.3)	0.8
Turkey offal or meat	0	3	0	0	3 (0.1)	0.3
Vegetables	0	1	0	0	1 (<0.1)	0.1
Wild bird	0	2	0	0	2 (0.1)	0.2
Total	939	1454	12	2	2407	100.0

ST, sequence type; SA, source attribution % excluding human isolates and isolates with no information; -, not included in the SA calculations.

**Table 5:** Source distribution of *Campylobacter* isolates by different genotypes based on the data mining analysis.

Source	Farm Animal	Pet	Poultry	Environment and Wild Bird	Human	Other Animal	Vegetable	No Information	Total
Genotype	Total Number (% of Row)								
ST22	220 (15.7)	12 (0.9)	154 (11.0)	14 (1.0)	636 (45.4)	19 (1.4)	0 (0.0)	345 (24.6)	1400
ST50	181 (3.6)	9 (0.2)	1752 (35.3)	22 (0.4)	2372 (47.8)	17 (0.3)	0 (0.0)	615 (12.4)	4968
ST122	6 (0.6)	7 (0.8)	189 (20.1)	3 (0.3)	669 (71.3)	3 (0.3)	0 (0.0)	62 (6.6)	939
ST353	17 (0.8)	5 (0.2)	1316 (62.6)	2 (0.1)	305 (14.5)	3 (0.1)	0 (0.0)	455 (21.7)	2103
ST429	0 (0.0)	0 (0.0)	41 (38.0)	3 (2.8)	47 (43.5)	0 (0.0)	0 (0.0)	17 (15.7)	108
ST464	16 (1.1)	1 (<0.1)	674 (46.4)	3 (0.2)	580 (39.9)	4 (0.3)	1 (<0.1)	175 (12.0)	1454
ST572	10 (1.4)	6 (0.8)	58 (8.1)	10 (1.4)	586 (81.6)	3 (0.4)	0 (0.0)	45 (6.3)	718
ST824	5 (1.5)	0 (0.0)	123 (37.5)	1 (0.3)	135 (41.2)	1 (0.3)	0 (0.0)	63 (19.2)	328
ST1595	6 (10.9)	0 (0.0)	14 (25.5)	3 (5.4)	31 (56.4)	0 (0.0)	0 (0.0)	1 (1.8)	55
ST1624	17 (70.8)	0 (0.0)	4 (16.7)	0 (0.0)	1 (4.2)	0 (0.0)	0 (0.0)	2 (8.3)	24
ST7355	0 (0.0)	0 (0.0)	1 (8.3)	0 (0.0)	9 (75.0)	0 (0.0)	0 (0.0)	2 (16.7)	12
ST9882	0 (0.0)	0 (0.0)	0 (0.0)	0 (0.0)	1 (50.0)	0 (0.0)	0 (0.0)	1 (50.0)	2
ST11001	0 (0.0)	0 (0.0)	0 (0.0)	0 (0.0)	0 (0.0)	0 (0.0)	0 (0.0)	0 (0.0)	0
Total	478 (4.0)	40 (0.3)	4326 (35.7)	61 (0.5)	5372 (44.4)	50 (0.4)	1 (<0.1)	1783 (14.7)	12,111
95% CI	3.6–4.3	0.2–0.5	34.9–36.6	0.4–0.7	43.5–45.3	0.3–0.6	0.0–0.1	14.1–15.4	

ST, sequence type; 95% CI, 95% confidence interval.

**Table 6:** Attribution (in percentages) of different *Campylobacter* genotypes to different sources, not accounting for human sources and sources with no information.

Genotype	Farm Animal	Pet	Poultry	Environment and Wild Bird	Other Animal	Vegetable
ST22	52.5	2.9	36.8	3.3	4.5	0.0
ST50	9.1	0.5	88.4	1.1	0.9	0.0
ST122	2.9	3.4	90.9	1.4	1.4	0.0
ST353	1.3	0.4	98.0	0.1	0.2	0.0
ST429	0.0	0.0	93.2	6.8	0.0	0.0
ST464	2.3	0.1	96.5	0.4	0.6	<0.1
ST572	11.5	6.9	66.7	11.5	3.4	0.0
ST824	3.8	0.0	94.6	0.8	0.8	0.0
ST1595	26.1	0.0	60.9	13.0	0.0	0.0
ST1624	81.0	0.0	19.0	0.0	0.0	0.0
ST7355	0.0	0.0	100.0	0.0	0.0	0.0
ST9882	0.0	0.0	0.0	0.0	0.0	0.0
ST11001	0.0	0.0	0.0	0.0	0.0	0.0
Total	9.7	0.8	87.3	1.2	1.0	<0.1

ST, sequence type.

**Table 7:** Disease outcomes by sequence type after confirmed *Campylobacter* spp. infection in humans.

ST	Carrier	Gastroenteritis	Guillain–Barré Syndrome	Systemic Disease	No Information	Total
22	5	366	25	2	238	636
50	3	1382	0	1	986	2372
122	0	364	1	1	303	669
353	7	175	0	2	121	305
429	0	21	0	0	26	47
464	0	303	0	0	277	580
572	0	277	0	0	309	586
824	0	71	0	0	64	135
1595	0	22	0	0	9	31
1624	0	1	0	0	0	1
7355	0	4	0	0	5	9
9882	0	0	0	0	1	1
11001	0	0	0	0	0	0
Total	15	2986	26	6	2339	5372

ST, sequence type.

**Table 8:** Epidemiological classification of human *Campylobacter* isolates belonging to MLST genotypes shared between Estonian clinical cases and broiler chicken meat, excluding 582 isolates with no information.

ST	Carrier	General Outbreak	Hospital Inpatient	Sporadic Case
ST122	0	0	1	348
ST464	2	5	3	316
ST7355	0	0	0	2
ST9882	0	0	0	0
Total (%)	2 (0.3)	5 (0.7)	4 (0.6)	666 (98.4)

ST, sequence type.

**Table 9:** Demographic characteristics (gender and age) of human *Campylobacter* isolates belonging to MLST genotypes shared between Estonian clinical cases and broiler chicken meat, excluding isolate data with no information on gender (*n* = 581) and age (*n* = 1040).

Gender/Age	ST122	ST464	ST7355	ST9882	Total
Female (%; 95% CI)	202 (50.2; 45.3–55.2)	119 (43.6; 37.7–49.7)	1 (33.3; 1.8–87.5)	0 (-; -)	322 (47.5; 43.7–51.3)
Male (%; 95% CI)	200 (49.8; 44.8–54.7)	154 (56.4; 50.3–62.3)	2 (66.7; 12.5–98.2)	0 (-; -)	356 (52.5; 48.7–56.3)
0–14 (average age)	22 (5.7)	12 (6.8)	1 (5.0)	0 (-)	35 (6.1)
15–64 (average age)	108 (36.1)	60 (36.7)	2 (35.5)	0 (-)	170 (36.3)
65–... (average age)	10 (70.6)	4 (70.0)	0 (-)	0 (-)	14 (70.4)
Total (average age)	140 (33.8)	76 (33.8)	3 (25.3)	0 (-)	219 (33.7)

ST, sequence type; 95% CI, 95% confidence interval; -, no data.

## Data Availability

The data were obtained from the publicly available open-access *C. jejuni*/*coli* PubMLST database.

## References

[1] EFSA (European Food Safety Authority), ECDC (European Centre for Disease Prevention and Control) (2023). The European Union One Health 2022 zoonoses report. EFSA J..

[2] EFSA (European Food Safety Authority), ECDC (European Centre for Disease Prevention and Control) (2024). The European Union One Health 2023 zoonoses report. EFSA J..

[3] Cody A.J., Maiden M.C., Strachan N.J., McCarthy N.D. (2019). A systematic review of source attribution of human campylobacteriosis using multilocus sequence typing. Euro Surveill..

[4] Garcia-Sanchez L., Melero B., Rovira L. (2018). *Campylobacter* in the food chain. Adv. Food Nutr. Res..

[5] Wilson D.J., Gabriel E., Leatherbarrow A.J., Cheesbrough J., Gee S., Bolton E., Fox A., Fearnhead P., Hart C.A., Diggle P.J. (2008). Tracing the source of campylobacteriosis. PLoS Genet..

[6] Little C.L., Gormley F.J., Rawal N., Richardson J.F. (2010). A recipe for disaster: Outbreaks of campylobacteriosis associated with poultry liver pâté in England and Wales. Epidemiol. Infect..

[7] Kaakoush N., Castaño-Rodríguez N., Mitchell H., Man S. (2015). Global epidemiology of *Campylobacter* infection. Clin. Microbiol. Rev..

[8] Mughini-Gras L., Penny C., Ragimbeau C., Schets F., Blaak H., Duim B., Wagenaar J., de Boer A., Cauchie H., Mossong J. (2016). Quantifying potential sources of surface water contamination with *Campylobacter jejuni* and *Campylobacter coli*. Water Res..

[9] Mäesaar M., Tedersoo T., Meremäe K., Roasto M. (2020). The source attribution analysis revealed the prevalent role of poultry over cattle and wild birds in human campylobacteriosis cases in the Baltic States. PLoS ONE.

[10] Ellis-Iversen J., Ridley A., Morris V., Sowa A., Harris J., Atterbury R., Sparks N., Allen V. (2012). Persistent environmental reservoirs on farms as risk factors for *Campylobacter* in commercial poultry. Epidemiol. Infect..

[11] Ahmed M.F., Schulz J., Hartung J. (2013). Survival of *Campylobacter jejuni* in naturally and artificially contaminated laying hen feces. Poult. Sci..

[12] Pitkänen T. (2013). Review of *Campylobacter* spp. in drinking and environmental waters. J. Microbiol. Methods.

[13] Turonova H., Haddad N., Hernould M., Chevret D., Pazlarova J., Tresse O. (2017). Profiling of *Campylobacter jejuni* proteome in exponential and stationary phase of growth. Front. Microbiol..

[14] Facciolà A., Riso R., Avventuroso E., Visalli G., Delia S.A., Laganà P. (2017). *Campylobacter*: From microbiology to prevention. J. Prev. Med. Hyg..

[15] EFSA (European Food Safety Authority) (2022). Story Map on *Campylobacter*. https://storymaps.arcgis.com/stories/37987745de6f47029e14cb57d61fe923.

[16] Meldrum R.J., Griffiths J.K., Smith R.M.M., Evans M.R. (2005). The seasonality of human *Campylobacter* infection and *Campylobacter* isolates from fresh, retail chicken in Wales. Epidemiol. Infect..

[17] Stafford R.J., Schluter P.J., Wilson A.J., Kirk M.D., Hall G., Unicomb L., OzFoodNet Working Group (2008). Population-attributable risk estimates for risk factors associated with *Campylobacter* infection, Australia. Emerg. Infect. Dis..

[18] Mughini Gras L., Smid J.H., Wagenaar J.A., de Boer A.G., Havelaar A.H., Friesema I.H., French N.P., Busani L., van Pelt W. (2012). Risk factors for campylobacteriosis of chicken, ruminant, and environmental origin: A combined case-control and source attribution analysis. PLoS ONE.

[19] Keener K.M., Bashor M.P., Curtis P.A., Sheldon B.W., Kathariou S. (2004). Comprehensive review of *Campylobacter* and poultry processing. Compr. Rev. Food Sci. Food Saf..

[20] Humphrey T., Martin K., Slader J., Durham K. (2001). *Campylobacter* spp. in the kitchen: Spread and persistence. J. Appl. Microbiol..

[21] Santos-Ferreira N., Alves Â., Cardoso M.J., Langsrud S., Malheiro A.R., Fernandes R., Maia R., Truninger M., Junqueira L., Nicolau A.I. (2021). Cross-contamination of lettuce with *Campylobacter* spp. via cooking salt during handling raw poultry. PLoS ONE.

[22] Blaeske V., Schumann-Muck F.M., Hamedy A., Braun P.G., Koethe M. (2024). *Campylobacter* colonisation of slaughterhouse surfaces may be affected by ultra-thin silica coating. AIMS Agric. Food.

[23] Dingle K.E., Colles F.M., Wareing D.R., Ure R., Fox A.J., Bolton F.E., Bootsma H.J., Willems R.J., Urwin R., Maiden M.C. (2001). Multilocus sequence typing system for *Campylobacter jejuni*. J. Clin. Microbiol..

[24] Koutsoumanis K., Allende A., Alvarez-Ordóñez A., Bolton D., Bover-Cid S., Chemaly M., Davies R., De Cesare A., Hilbert F., EFSA BIOHAZ Panel (EFSA Panel on Biological Hazards) (2019). Whole genome sequencing and metagenomics for outbreak investigation, source attribution and risk assessment of food-borne microorganisms. EFSA J..

[25] Cody A.J., Bray J.E., Jolley K.A., McCarthy N.D., Maiden M.C.J. (2017). Core genome multilocus sequence typing scheme for stable, comparative analyses of *Campylobacter jejuni* and *C. coli* human disease isolates. J. Clin. Microbiol..

[26] Nennig M., Llarena A.-K., Herold M., Mossong J., Penny C., Losch S., Tresse O., Ragimbeau C. (2021). Investigating major recurring *Campylobacter jejuni* lineages in Luxembourg using four core or whole genome sequencing typing schemes. Front. Cell. Infect. Microbiol..

[27] Llarena A.K., Taboada E., Rossi M. (2017). Whole-genome sequencing in epidemiology of *Campylobacter jejuni* infections. J. Clin. Microbiol..

[28] Joensen K.G., Kiil K., Gantzhorn M.R., Nauerby B., Engberg J., Holt H.M., Nielsen H.L., Petersen A.M., Kuhn K.G., Sandø G. (2020). Whole-genome sequencing to detect numerous *Campylobacter jejuni* outbreaks and match patient isolates to sources, Denmark, 2015–2017. Emerg. Infect. Dis..

[29] Joensen K.G., Schjørring S., Gantzhorn M.R., Vester C.T., Nielsen H.L., Engberg J.H., Holt H.M., Ethelberg S., Müller L., Sandø G. (2021). Whole genome sequencing data used for surveillance of *Campylobacter* infections: Detection of a large continuous outbreak, Denmark, 2019. Euro Surveill..

[30] Pires S.M., Evers E.G., van Pelt W., Ayers T., Scallan E., Angulo F.J., Havelaar A., Hald T., Med-Vet-Net Workpackage 28 Working Group (2009). Attributing the human disease burden of foodborne infections to specific sources. Foodborne Pathog. Dis..

[31] Hald T. (2013). Chapter 5—Pathogen updates: *Salmonella*. Foodborne Infections and Intoxications.

[32] Arning N., Sheppard S., Bayliss S., Clifton D., Wilson D. (2021). Machine learning to predict the source of campylobacteriosis using whole genome data. PLoS Genet..

[33] Tedersoo T., Roasto M., Mäesaar M., Kisand V., Ivanova M., Meremäe K. (2022). The prevalence, counts, and MLST genotypes of *Campylobacter* in poultry meat and genomic comparison with clinical isolates. Poult. Sci..

[34] Jolley K.A., Bray J.E., Maiden M. (2018). Open-access bacterial population genomics: BIGSdb software, the PubMLST.org website and their applications. Wellcome Open Res..

[35] Jolley K.A., Maiden M.C. (2010). BIGSdb: Scalable analysis of bacterial genome variation at the population level. BMC Bioinform..

[36] Camacho C., Coulouris G., Avagyan V., Ma N., Papadopoulos J., Bealer K., Madden T.L. (2009). BLAST+: Architecture and applications. BMC Bioinform..

[37] Bortolaia V., Kaas R.S., Ruppe E., Roberts M.C., Schwarz S., Cattoir V., Philippon A., Allesoe R.L., Rebelo A.R., Florensa A.F. (2020). ResFinder 4.0 for predictions of phenotypes from genotypes. J. Antimicrob. Chemother..

[38] Wilson E.B. (1927). Probable inference, the law of succession, and statistical inference. J. Am. Stat. Assoc..

[39] Newcombe R.G. (1998). Two-sided confidence intervals for the single proportion: Comparison of seven methods. Stat. Med..

[40] (2025). VassarStats. Website for Statistical Computation. http://vassarstats.net/prop1.html.

[41] Tedersoo T., Roasto M., Mäesaar M., Häkkinen L., Kisand V., Ivanova M., Valli M.H., Meremäe K. (2022). Antibiotic resistance in *Campylobacter* spp. isolated from broiler chicken meat and human patients in Estonia. Microorganisms.

[42] Igwaran A., Okoh A.I. (2019). Human campylobacteriosis: A public health concern of global importance. Heliyon.

[43] Hansson I., Sandberg M., Habib I., Lowman R., Engvall E.O. (2018). Knowledge gaps in control of *Campylobacter* for prevention of campylobacteriosis. Transbound. Emerg. Dis..

[44] Kuhn K.G., Nielsen E.M., Mølbak K., Ethelberg S. (2018). Determinants of sporadic *Campylobacter* infections in Denmark: A nationwide case-control study among children and young adults. Clin. Epidemiol..

[45] Thystrup C., Brinch M.L., Henri C., Mughini-Gras L., Franz E., Wieczorek K., Gutierrez M., Prendergast D.M., Duffy G., Burgess C.M. (2025). Source attribution of human *Campylobacter* infection: A multi-country model in the European Union. Front. Microbiol..

[46] Wieczorek K., Wołkowicz T., Osek J. (2020). MLST-based genetic relatedness of *Campylobacter jejuni* isolated from chickens and humans in Poland. PLoS ONE.

[47] Mossong J., Mughini-Gras L., Penny C., Devaux A., Olinger C., Losch S., Cauchie H.M., van Pelt W., Ragimbeau C. (2016). Human campylobacteriosis in Luxembourg, 2010–2013: A case-control study combined with multilocus sequence typing for source attribution and risk factor analysis. Sci. Rep..

[48] Guyard-Nicodème M., Rivoal K., Houard E., Rose V., Quesne S., Mourand G., Rouxel S., Kempf I., Guillier L., Gauchard F. (2015). Prevalence and characterization of *Campylobacter jejuni* from chicken meat sold in French retail outlets. Int. J. Food Microbiol..

[49] Stevens M.J.A., Stephan R., Horlbog J.A., Cernela N., Nüesch-Inderbinen M. (2024). Whole genome sequence-based characterization of *Campylobacter* isolated from broiler carcasses over a three-year period in a big poultry slaughterhouse reveals high genetic diversity and a recurring genomic lineage of *Campylobacter jejuni*. Infect. Genet. Evol..

[50] Strachan N.J., Watson R.O., Novik V., Hofreuter D., Ogden I.D., Galán J.E. (2008). Sexual dimorphism in campylobacteriosis. Epidemiol. Infect..

[51] Green M.S., Schwartz N., Peer V. (2020). Sex differences in campylobacteriosis incidence rates at different ages—A seven country, multi-year, meta-analysis. A potential mechanism for the infection. BMC Infect. Dis..

[52] Mandel T., Condoleo R., Zarea A.A.K., Mäesaar M., Roasto M., Reinik M., Iulietto M.F. (2026). Fluoroquinolone resistance in *Campylobacter* isolates from broiler meat: A systematic review and meta-analysis at a global scale. Food Rev. Int..

[53] ECDC (European Centre for Disease Prevention and Control), EFSA (European Food Safety Authority), EMA (European Medicines Agency) (2024). Fourth joint inter-agency report on integrated analysis of antimicrobial consumption and occurrence of antimicrobial resistance in bacteria from humans and food-producing animals in the EU/EEA (2019–2021). EFSA J..

[54] Mäesaar M., Kramarenko T., Meremäe K., Sõgel J., Lillenberg M., Häkkinen L., Ivanova M., Kovalenko K., Hörman A., Hänninen M.-L. (2016). Antimicrobial resistance profiles of *Campylobacter* spp. isolated from broiler chicken meat of Estonian, Latvian and Lithuanian origin at Estonian retail level and from patients with severe enteric infections in Estonia. Zoonoses Public Health.

[55] Mäesaar M., Meremäe K., Ivanova M., Roasto M. (2018). Antimicrobial resistance and multilocus sequence types of *Campylobacter jejuni* isolated from Baltic broiler chicken meat and Estonian human patients. Poult. Sci..

[56] Aksomaitiene J., Ramonaite S., Tamuleviciene E., Novoslavskij A., Alter T., Malakauskas M. (2019). Overlap of antibiotic resistant *Campylobacter jejuni* MLST genotypes isolated from humans, broiler products, dairy cattle and wild birds in Lithuania. Front. Microbiol..

[57] Meistere I., Ķibilds J., Eglīte L., Alksne L., Avsejenko J., Cibrovska A., Makarova S., Streikiša M., Grantiņa-Ieviņa L., Bērziņš A. (2019). *Campylobacter* species prevalence, characterisation of antimicrobial resistance and analysis of whole-genome sequence of isolates from livestock and humans, Latvia, 2008 to 2016. Euro Surveill..

[58] WHO (World Health Organization) (2017). The Burden of Foodborne Diseases in the WHO European Region. https://www.quotidianosanita.it/allegati/allegato6840262.pdf.

[59] Rao M.R., Naficy A.B., Savarino S.J., Abu-Elyazeed R., Wierzba T.F., Peruski L.F., Abdel-Messih I., Frenck R., Clemens J.D. (2001). Pathogenicity and convalescent excretion of *Campylobacter* in rural Egyptian children. Am. J. Epidemiol..

[60] Jackson B.R., Zegarra J.A., López-Gatell H., Sejvar J., Arzate F., Waterman S., Núñez A.S., López B., Weiss J., Cruz R.Q. (2014). Binational outbreak of Guillain-Barré syndrome associated with *Campylobacter jejuni* infection, Mexico and USA, 2011. Epidemiol. Infect..

[61] Robinson D.A. (1981). Infective dose of *Campylobacter jejuni* in milk. BMJ.

[62] Johnson T.J., Shank J.M., Johnson J.G. (2017). Current and potential treatments for reducing *Campylobacter* colonization in animal hosts and disease in humans. Front. Microbiol..

[63] Ramonaite S., Kudirkiene E., Tamuleviciene E., Leviniene G., Malakauskas A., Gölz G., Alter T., Malakauskas M. (2014). Prevalence and genotypes of *Campylobacter jejuni* from urban environmental sources in comparison with clinical isolates from children. J. Med. Microbiol..

[64] Ramonaite S., Tamuleviciene E., Alter T., Kasnauskyte N., Malakauskas M. (2017). MLST genotypes of *Campylobacter jejuni* isolated from broiler products, dairy cattle and human campylobacteriosis cases in Lithuania. BMC Infect. Dis..

[65] Weinberger M., Moran-Gilad J., Rokney A., Davidov Y., Agmon V., Peretz C., Valinsky L. (2016). Molecular epidemiology of *Campylobacter jejuni* infection in Israel—A nationwide study. Clin. Microbiol. Infect..

[66] Reichelt B., Szott V., Epping L., Semmler T., Merle R., Roesler U., Friese A. (2022). Transmission pathways of *Campylobacter* spp. at broiler farms and their environment in Brandenburg, Germany. Front. Microbiol..

[67] Šoprek S., Duvnjak S., Kompes G., Jurinović L., Tambić Andrašević A. (2022). Resistome analysis of *Campylobacter jejuni* strains isolated from human stool and primary sterile samples in Croatia. Microorganisms.

[68] Mäesaar M., Mamede R., Elias T., Roasto M. (2021). Retrospective use of whole-genome sequencing expands the multicountry outbreak cluster of *Listeria monocytogenes* ST1247. Int. J. Genom..

[69] Wilson D.J., Gabriel E., Leatherbarrow A.J., Cheesbrough J., Gee S., Bolton E., Fox A., Hart C.A., Diggle P.J., Fearnhead P. (2009). Rapid evolution and the importance of recombination to the gastroenteric pathogen *Campylobacter jejuni*. Mol. Biol. Evol..

[70] Frosth S., Karlsson-Lindsjö O., Niazi A., Fernström L.L., Hansson I. (2020). Identification of transmission routes of *Campylobacter* and on-farm measures to reduce *Campylobacter* in chicken. Pathogens.

[71] Heikema A.P., Islam Z., Horst-Kreft D., Huizinga R., Jacobs B.C., Wagenaar J.A., Poly F., Guerry P., van Belkum A., Parker C.T. (2015). *Campylobacter jejuni* capsular genotypes are related to Guillain-Barré syndrome. Clin. Microbiol. Infect..

[72] Sheppard S.K., Dallas J.F., MacRae M., McCarthy N.D., Sproston E.L., Gormley F.J., Strachan N.J., Ogden I.D., Maiden M.C., Forbes K.J. (2009). *Campylobacter* genotypes from food animals, environmental sources and clinical disease in Scotland 2005/6. Int. J. Food Microbiol..

[73] Nohra A., Grinberg A., Midwinter A.C., Marshall J.C., Collins-Emerson J.M., French N.P. (2016). Molecular epidemiology of *Campylobacter coli* strains isolated from different sources in New Zealand between 2005 and 2014. Appl. Environ. Microbiol..

[74] Rosner B.M., Schielke A., Didelot X., Kops F., Breidenbach J., Willrich N., Gölz G., Alter T., Stingl K., Josenhans C. (2017). A combined case-control and molecular source attribution study of human *Campylobacter* infections in Germany, 2011–2014. Sci. Rep..

[75] Wieczorek K., Osek J. (2013). Antimicrobial resistance mechanisms among *Campylobacter*. BioMed Res. Int..

